# Enniatin Production Influences *Fusarium avenaceum* Virulence on Potato Tubers, but not on Durum Wheat or Peas

**DOI:** 10.3390/pathogens9020075

**Published:** 2020-01-21

**Authors:** Anas Eranthodi, Danielle Schneiderman, Linda J. Harris, Thomas E. Witte, Amanda Sproule, Anne Hermans, David P. Overy, Syama Chatterton, Jiajun Liu, Tao Li, Dianevys González-Peña Fundora, Weiquan Zhao, Nora A. Foroud

**Affiliations:** 1 Lethbridge Research and Development Centre, Agriculture and Agri-Food Canada, 5403–1st Avenue South, Lethbridge, AB T1J 4B1, Canada; anas.eranthodi@canada.ca (A.E.); syama.chatterton@canada.ca (S.C.); dianevys.gonzalezpenafundora@canada.ca (D.G.-P.F.); 2 Ottawa Research and Development Centre, Agriculture and Agri-Food Canada, 960 Carling Avenue, Central Experimental Farm, Ottawa, ON K1A 0C6, Canada; danielle.schneiderman@canada.ca (D.S.); linda.harris@canada.ca (L.J.H.); tom.witte@canada.ca (T.E.W.); amanda.sproule@canada.ca (A.S.); anne.hermans@canada.ca (A.H.); david.overy@canada.ca (D.P.O.); 3 Department of Crop Genetics and Breeding and Applied Biotechnology, Yangzhou University, 12 Wenhui East Road, Yangzhou 225009, China; divergent9304@hotmail.com (J.L.); taoli@yzu.edu.cn (T.L.); 4 Department of Plant Pathology, Hebei Agricultural University, NO.2596 Lekai South Street, Baoding 071000, China; zhaowquan@126.com

**Keywords:** enniatins, mycotoxins, *Fusarium*, Fusarium head blight, Fusarium root rot, durum wheat, pea, potato, virulence

## Abstract

*Fusarium avenaceum* is a generalist pathogen responsible for diseases in numerous crop species. The fungus produces a series of mycotoxins including the cyclohexadepsipeptide enniatins. Mycotoxins can be pathogenicity and virulence factors in various plant–pathogen interactions, and enniatins have been shown to influence aggressiveness on potato tubers. To determine the role of these mycotoxins in other *F. avenaceum*–host interactions, *ENNIATIN SYNTHASE* 1 (*ESYN*1) disruption and overexpression mutants were generated and their ability to infect wheat and peas investigated. As a preliminary study, the transformants were screened for their ability to cause potato tuber necrosis and, consistent with a previous report, enniatin production increased necrotic lesion size on the tubers. By contrast, when the same mutants were assessed in their ability to cause disease in pea roots or durum wheat spikes, no changes in disease symptoms or virulence were observed. While it is known that, at least in the case of wheat, exogenously applied enniatins can cause tissue necrosis, this group of mycotoxins does not appear to be a key factor on its own in disease development on peas or durum wheat.

## 1. Introduction

*Fusarium avenaceum* is a generalist pathogen involved in a wide variety of plant diseases, including Fusarium root and stalk rots of diverse crops species [1,2,3], and Fusarium head blight (FHB) of cereals [4]. Potatoes, peas, and cereals are among the major agricultural crops affected by this species. Fusarium dry rot is an important disease of potatoes worldwide [5], with potential crop losses resulting from tuber dry rot in the field and in storage in the neighborhood of 25% and 60%, respectively [6,7,8]. Removal of damaged tubers prior to storage can help to reduce the impact of this disease, as the fungus readily infects wounded or bruised tubers and proliferates slowly in storage [9]. Symptoms include dark depressions on the tuber surface, which turn into larger lesions of wrinkled skin over necrotic areas [7]. Different *Fusarium* mycotoxins, such as enniatins, beauvericin, and trichothecenes, have been found to accumulate in infected potato tissues and some of these may be involved in pathogenicity [10,11,12,13]. *F. avenaceum* is among the top four *Fusarium spp.* commonly associated with the disease; although, the predominant species varies according to geographic and temporal parameters [6,14,15,16]. 

Pea root rot is caused by a group of pathogens involving multiple genera of fungi, including *Fusarium*, *Alternaria,* and *Peyronellaea* spp. as well as the oomycete pathogen, *Aphanomyces euteiches* [1,17], and is the largest biotic constraint to pea production in North America [18]. While a number of *Fusarium* species, such as *F. solani* and *F. redolens*, are associated with the disease, *F. avenaceum* has become increasingly linked to pea root rot in recent years. It was the dominant fungus identified in 70–90% of pea fields in recent surveys in the Canadian prairies and North Dakota [19,20], and was routinely isolated from pea roots in Europe [21,22]. Among the *Fusarium* species isolated from pea roots, isolates of *F. avenaceum* were the most virulent on pea, although there was significant variation in virulence [20,21,23]. Root rot symptoms caused by *F. avenaceum* include red-brown lesions on the tap root, starting at the point of seed attachment [1]. Progressive elongation of the lesions along the tap root can result in blackening of the tap root, often accompanied by reddening of the vascular system and death of the plant. Although *F. avenaceum* is an important pathogen of pea, there have been no studies on mycotoxin production by this fungus on pea.

Different tissues of cereals can be infected by *Fusarium* species [24,25,26], but FHB, a disease of the inflorescence structure, has worldwide economic significance [25]. The fungus infects the spike during flowering and grain development stages, and the resulting mycotoxin contamination of the kernels represent significant economic damage [27] since mycotoxins are harmful to human and animal consumers [28,29]. The trichothecene mycotoxins, such as deoxynivalenol (DON), were found to be important factors of aggressiveness in the *F. graminearum* interaction with the wheat spike [30]. While the main species responsible for FHB worldwide belong to the *F. graminearum* species complex, *F. avenaceum* is frequently isolated from diseased heads of cereals throughout Europe and North America [31,32,33,34]. In some Northern European countries, *F. avenaceum* is the dominant species identified from spikes of wheat, barley, and oats [34]. Gräfenhan et al. [32] reported that *F. avenaceum* and *F. graminearum* were the most frequently isolated species from the heads of wheat and rye in Western Canada in 2010. From the same survey, mycotoxin profiling by Tittlemier et al. [35] revealed that *F. avenaceum* enniatin (ENN) toxins were present in all of the durum wheat samples collected, whereas the trichothecene mycotoxin, DON, commonly found in FHB infected wheat, was detected in 75% of the samples. Despite their prevalence, the ENNs are not yet included in the standard screening practices for grain products; however, the regulatory status of ENNs are currently being evaluated in the EU as a result of the increasing evidence of their incidence in food and feed [36].

Mycotoxins often play critical roles in plant–pathogen interactions [12,30,37,38]. It is a logical assumption that necrosis-inducing mycotoxins in *F. avenaceum* would contribute to their interaction with different plant species. While *F. avenaceum* does not produce trichothecenes, genomic analysis indicates that this species is capable of producing a wide range of secondary metabolites [39], and indeed numerous metabolites have been identified from these fungi including enniatins (ENNs), beauvericin, and moniliformin [39,40]. ENNs are a group of cyclohexadepsipeptide toxins [41] first identified in cultures of *F. oxysporum* (previously described as *Fusarium orthoceras* var. *enniatum*) (reviewed in [29]). Cyclohexadepsipeptides complex with metal cations often forming ionophores in biological membranes [42], which is thought to be the main mechanism of ENNs toxicity [43]. The ionophores alter cation gradients across cell, thereby affecting cellular homeostasis. Toxicity testing in mice suggest that ENNs are both genotoxic and embryotoxic [44,45]. *F. avenaceum* produces at least 14 known ENN analogs, where ENN B, B1, A, and A1 are the most frequently detected ENNs from silage and inoculated maize [46,47]. ENN production was previously shown to enhance necrosis and *F. avenaceum* aggressiveness in potato tubers [11,12]. It has been observed that exogenous application of ENNs can inhibit growth of wheat seedling [48], but the role of ENNs in the FHB-wheat interaction has not been explored. Furthermore, the role of mycotoxin production has not been examined in root rot of pulse crops. In this study, the *ENNIATIN SYNTHASE 1* (*ESYN1*) gene was targeted in two *F. avenaceum* isolates for gene disruption or overexpression, and studied their interaction with potato tubers, pea roots, and the inflorescence of both common and durum wheat species.

## 2. Results

### 2.1. Generation of F. avenaceum ESYN1 Disruption and Overexpression Strains

**Figure 1:**
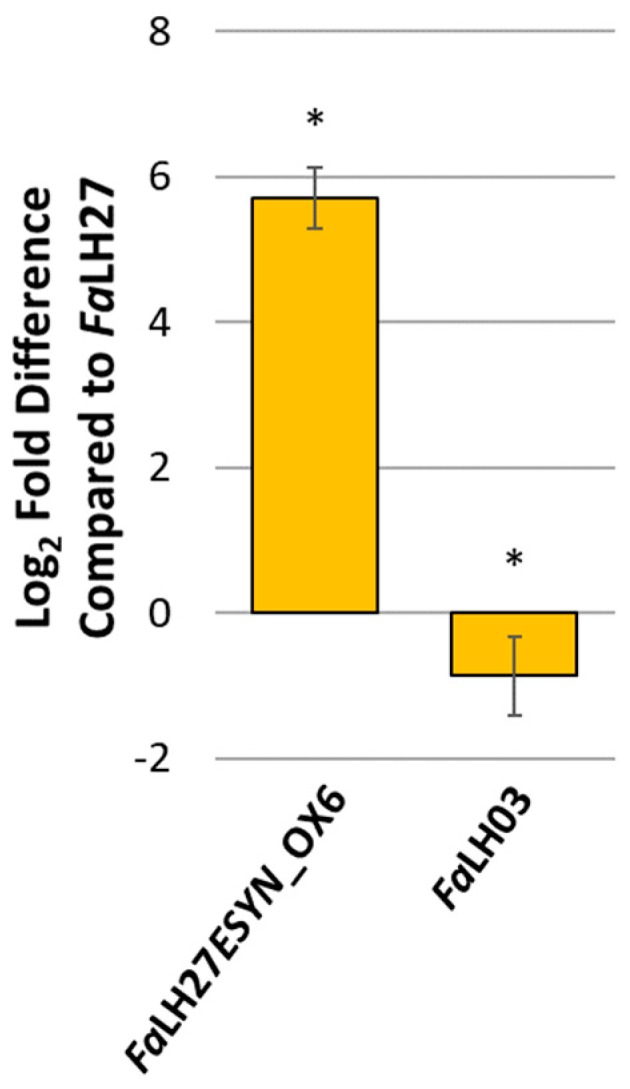
Fold difference for *ESYN1* gene expression compared with *Fa*LH27. Relative expression and difference (* *p* < 0.001) were calculated using Qiagen’s REST© software.

Three *ESYN1* disruption mutants (∆*esyn1)* were prepared in the wild-type (WT) background *Fa*LH03 (*Fa*LH03∆*esyn1*_2.1, *Fa*LH03∆*esyn1*_5.1, and *Fa*LH03∆*esyn1*_6.1) and two in WT *Fa*LH27 (*Fa*LH27∆*esyn1*_2 and *Fa*LH27∆*esyn1*_8) as well as one constitutive overexpression (*ESYN1*_OX) strain (*Fa*LH27*ESYN1*_OX6). All mutants carry a single copy of the hygromycin selection marker, *HYGROMYCIN PHOSPHOTRANSFERASE* (*HPH*) (Figure S1). For each of the disruption mutants, Southern blot analysis (Figure S1) confirmed the absence of the *ESYN1* gene. For the overexpression strain, only a single copy of the *ESYN1* gene was detected, and the relative expression of this gene was 5.71-fold higher (log_2_) (Figure 1) in *Fa*LH27*ESYN1*_OX6 compared with its WT progenitor, *Fa*LH27. Together the copy number and expression data indicate successful in locus overexpression of the ENN synthase gene in *Fa*LH27*ESYN1*_OX6. 

### 2.2. Genetic Disruption of ESYN1 Has a Small Positive Effect on Some Fungal Growth Parameters

**Figure 2:**
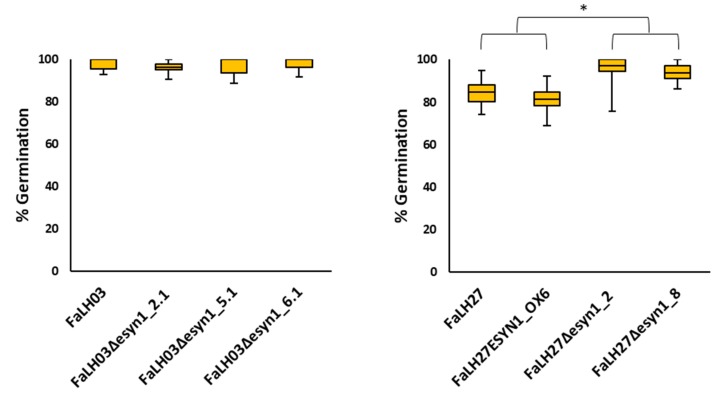
Box plots of spore germination tests for *Fa*LH03 and *Fa*LH27 and derived ∆*esyn1* and *ESYN1_OX* isolates. Percent germination was calculated 20 h after incubation of spores on SNA agar plates. Data were analyzed using ANOVA and means separated by Fisher’s least significant difference (LSD). * *p* < 0.05.

**Figure 3:**
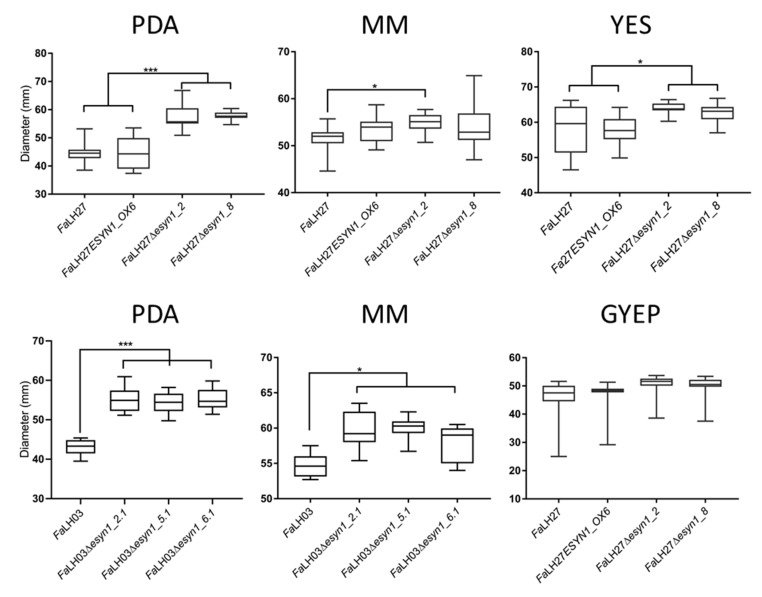
Box plots of growth assays of *Fa*LH03, *Fa*LH27 and derived ∆*esyn1* and *ESYN1_OX* isolates on different media. Radial growth measured on Day 5 and one-way ANOVA was used for Tukey multiple comparisons tests. * *p* < 0.05; *** *p* < 0.001.

Spore germination and mycelial growth assays were carried out to determine whether *ESYN1* disruption or overexpression could affect the general fitness of *F. avenaceum*. No significant effect on spore germination was observed among the *Fa*LH03 WT or genetically modified isolates thereof (Figure 2). By contrast, germination was lower in the *Fa*LH27 WT as well as the *ESYN* overexpression strain compared with either disruption mutant. Mycelial growth assays were carried out for *Fa*LH27 and derived isolates on petri dishes with potato dextrose agar (PDA), minimal media (MM), yeast extract sucrose (YES), or glucose yeast extract peptone (GYEP). For *Fa*LH03, the assays were carried out on PDA and MM. Under most conditions, an increase in mycelial growth rate was observed for the *ESYN1* disruption mutants compared with the WT progenitors (Figure S2 and Figure 3). No significant differences were observed between *Fa*LH27 and disruption mutants grown on GYEP (Figure 3).

### 2.3. Metabolic Analysis of Wild-type, ∆esyn1, and ESYN1_OX Isolates

**Figure 4:**
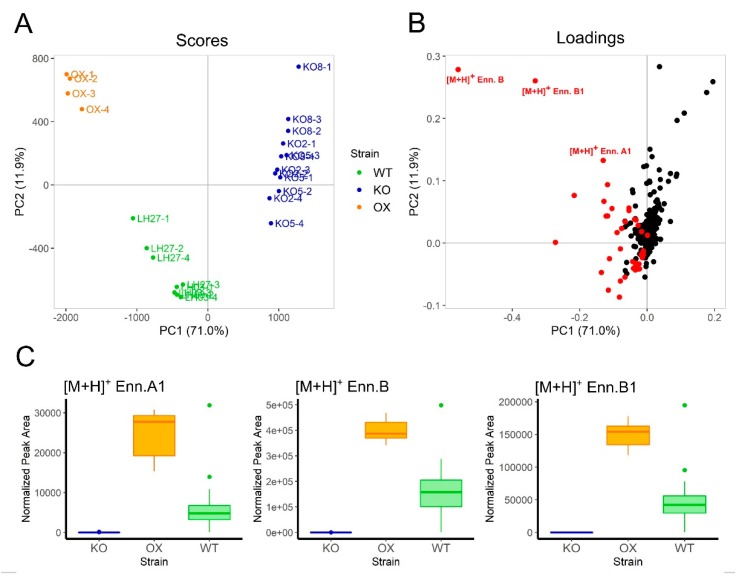
(**A**) PCA score plot comparing representative summed variable area secondary metabolite phenotypes produced from *Fa*LH27 and *Fa*LH03 (WT), Δ*esyn1* (KO), and *ESYN1_OX* (OX) strains cultured on multiple media (*n* = 4 replicates). Cumulative proportion of variance explained in first two principle components is 82.9%. (**B**) Loadings plot of first two PCs from the same PCA. Red dots represent variable features annotated as ENN-associated pseudo-molecular ions (the three most abundant ENN-associated ions are annotated in text). (**C**) Box plots comparing normalized peak areas of the [M + H]^+^ ion of the most abundant ENNs (A1, B, and B1) associated with WT, Δ*esyn1* (KO), and *ESYN1_OX* strains grown in all media types (data not summed).

Secondary metabolite profiling by Ultra-high Performance Liquid Chromatography – High Resolution Mass Spectrometry / Diode Array Detection / Charged Aerosol-spray Detection (UPLC-HRMS/DAD/CAD) was carried out using solvent extracts for *Fa*LH27 WT and progenitor Δ*esyn1* and *ESYN1_*OX strains, as well as *Fa*LH03 WT and progenitor Δ*esyn1* strains, cultured on a variety of media. A summation data matrix of pseudomolecular ion peaks derived from UPLC-HRMS metabolite profiles was compiled for each strain grown on representative media conditions and a PCA analysis was performed to visualize the impact of *ESYN1* disruption or overexpression on secondary metabolite production compared to WT. A clear separation was observed of *Fa*LH27 and *Fa*LH03 Δ*esyn1* strains (KO) from that of WT and *ESYN1_*OX (OX) strains along PC1 (Figure 4A), which accounted for 71% of the explained variance of the data model. Variables associated with the production of ENNs (highlighted in red, Figure 4B; ENN annotations assigned through commercial standards and targeted MS/MS experiments) were observed to be the greatest contributors to the data model variance associated with the WT strains (*Fa*WT) and *ESYN1_*OX6 (negative loadings along PC1) compared with Δ*esyn1* mutants. Separation of *ESYN1_*OX6 from *Fa*WT strains was observed along PC2 (12% of explained variance), with [M + H]^+^ variables associated with ENNs B, B1, and A1 having the greatest positive loadings on PC2 associated with the *ESYN1_*OX strain. From a pairwise comparison, relative production of ENNs B, B1, and A1 were found to be significantly greater (*p* < 0.05) in *ESYN1_*OX6 compared to *Fa*WT (based on normalized [M + H]^+^ ion peak area; Figure 4C) while production was absent in *Fa*LH27 and *Fa*LH03 Δ*esyn1* strains on all media. 

### 2.4. Impact of ESYN1 Disruption or Overexpression on F. avenaceum Aggressiveness in Different Field Crops

#### 2.4.1. Potato Tuber Necrosis is Increased in the *ESYN1* Overexpression Isolate and Reduced in the Genetic Disruption Mutants

**Figure 5:**
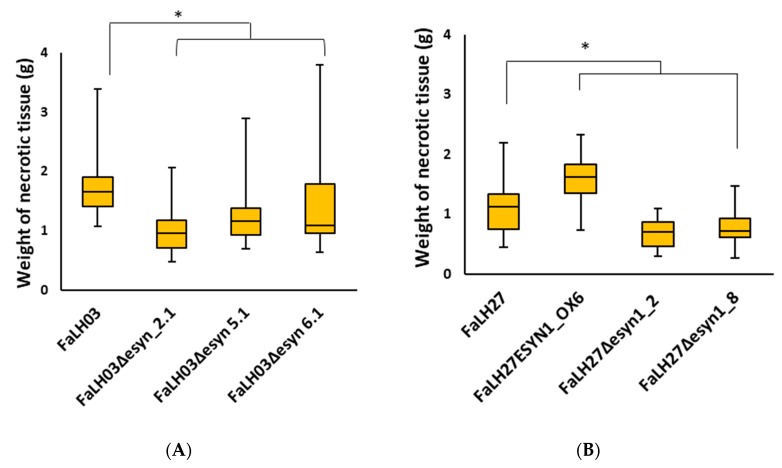
Box plots of potato tuber necrosis assays using (**A**) *Fa*LH03 and (**B**) *Fa*LH27 and derived ∆*esyn1* and *ESYN1_OX* isolates. The amount of necrosis was calculated six days after incubation of tuber slices. Data were analyzed using ANOVA and means separated by Fisher’s least significant difference (LSD). * *p* < 0.05

Potato tuber necrosis experiments were performed in Russet Burbank, the predominant commercial potato variety grown in North America, to verify the role of enniatin production on the aggressiveness of *F. avenaceum* isolates on potato tubers. Inoculation of potato tuber slices with ∆*esyn1* mutants of *Fa*LH03 and *Fa*LH27 isolates resulted in reduced necrosis on tubers, while the opposite was observed for *FaLH27ESYN1_OX6* when compared to its progenitor strain, *Fa*LH27 (Figure 5 and Figure S3). 

#### 2.4.2. No Difference Was Observed in FHB Disease Spread Among *ESYN1*-Modified Isolates and Wild-Type *F. avenaceum*

**Figure 6:**
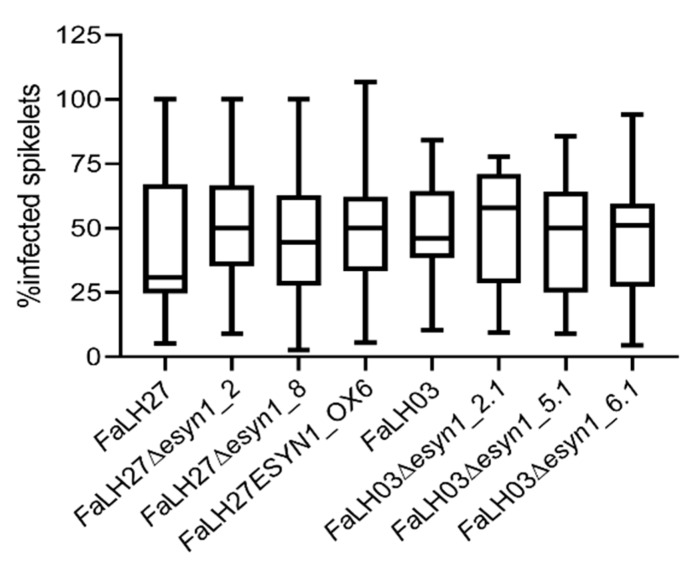
Box plots of FHB assays for *Fa*LH03, *Fa*LH27 and derived ∆*esyn1* and *ESYN1_OX* isolates in durum wheat cultivar Langdon. Percent of infected spikelets was measured 14 days after point inoculation. The experiment was repeated three times with 10–15 replicates per treatment. Data were analyzed using a one-way ANOVA, and showed no significant difference in virulence among isolates.

To determine whether altered ENN production influences pathogenicity of *F. avenaceum* in durum wheat, point inoculation experiments were carried out in the FHB moderately susceptible cultivar Langdon. Langdon has been commonly used by breeders to introduce and map traits including FHB resistance from related wild wheat species into durum wheat [49]. Disease symptoms were rated over the course of 14 days by counting the number of infected spikelets, and the results at 14 days is presented in Figure 6. No difference in aggressiveness was observed among the WT strains and the *ESYN1* overexpression and disruption mutants. In contrast with durum wheat, *Fa*LH03 and *Fa*LH27 only cause infection in the inoculated spikelet in hexaploid wheat (*Triticum aestivum* L.). To determine whether *ESYN1* overexpression would enable the fungus to spread in the spikes of hexaploid wheat, a point inoculation study with *Fa*LH27 and the *ESYN1* overexpression isolate *Fa*LH27*ESYN1*_OX6 was carried out in the highly susceptible hexaploid wheat cultivar Roblin. The infection was contained in the inoculated Roblin spikelets regardless of the isolate used (data not shown).

#### 2.4.3. No Difference Was Observed in Pea Emergence or Root Rot Symptoms Among *ESYN1*-modified Isolates and Wild-type *F. avenaceum*

Seeds of susceptible (cv. CDC Meadow) and partially resistant (cv. CDC Dakota) pea cultivars were inoculated with *F. avenaceum* WT and mutant strains and planted in vermiculite. These cultivars were chosen based on their differential response in greenhouse screening of pea germplasm lines and cultivars for *F. avenaceum* resistance for the pea breeding program at AAFC (Chatterton, data not published). Pea seeds, particularly cv. CDC Meadow, exhibited very poor emergence when inoculated with *F. avenaceum* and any of the mutants (Table 1). Mock-inoculated seeds had germination percentages ranging from 75 to 100%, demonstrating good overall seed viability, but were not included in the statistical analysis for the purpose of comparing mutants to WT. There were differences in emergence between repeated trials, and the interaction between cultivar and strain was significant; thus, for each trial, cultivar and isolate were analyzed separately. There was no significant difference between percent emergence after inoculation with WT and derived mutants, except for Trial 1 when Dakota was inoculated with *Fa*LH27 and mutants. Inoculation with one of the disruption mutants, *Fa*LH27Δ*esyn1*_8, resulted in a significantly higher percent emergence compared to the WT, but it was not significantly different from the other mutants (Table 1). 

**Figure 7:**
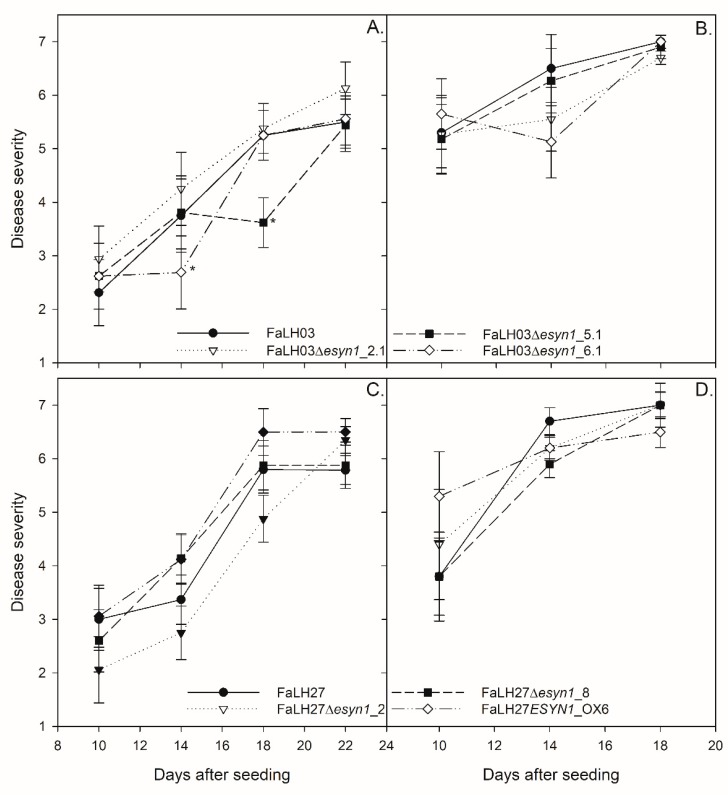
Pea root rot assays for *Fa*LH03 (**A**,**B**), *Fa*LH27 (**C**,**D**) and derived ∆*esyn1* and *ESYN1_OX* isolates in pea cultivars CDC Dakota (**A**,**C**) and CDC Meadow (**B**,**D**). Root rot severity was rated on a 1 (no disease) to 7 (dead plant) scale at 10, 14, 18, and 21 days after seeding. The experiment was performed two times with four replicates per treatment per day. (*) indicates significant difference (*p* < 0.05) between mutant and WT.

Root rot severity was rated every four days starting 10 days after seeding (DAS) (Figure 7). Root rot disease symptoms (DS) were fairly low (DS = 2–3) at 10 DAS for cv. CDC Dakota, but were already very high for cv. CDC Meadow (DS = 5–6). By 21 DAS, root rot levels on cv. CDC Dakota had progressed to 6–7, indicating complete necrosis of the root and death of the plant. These levels occurred on cv. CDC Meadow by 14 DAS, and by 21 DAS there was no root material remaining and thus roots could not be rated or kept for enniatin analysis. There was no consistent significant difference between the WT and mutants for any cultivar-strain combination. At 14 DAS, *Fa*LH03Δ*esyn1*_6.1 had a significantly lower root rating than the WT, as did *Fa*LH03Δ*esyn1*_5.1 at 18 DAS. However, significant differences between wild-types and derived mutants were not observed at any other time points.

**Figure 8:**
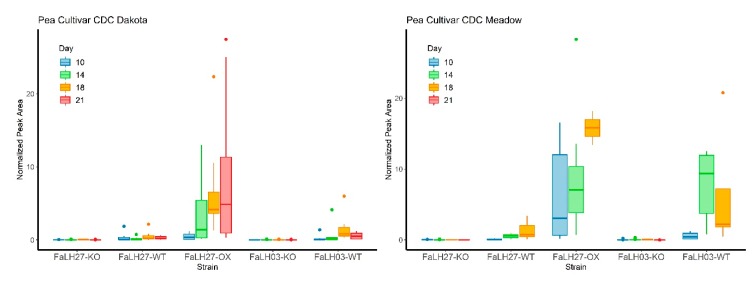
Box plots comparing relative peak areas of ENN B [M + H]^+^ ion detected from infected tissue extracts sampled from pea cultivars CDC Dakota (left) and CDC Meadow (right) at Days 10, 14, 18, and 21 days after seeding with *Fa*LH27, *Fa*LH03 WT, Δ*esyn1* (KO), and *ESYN1_OX* strains. Peak areas were normalized to caffeine internal standard added to each sample. Dots above box plots represent outliers, while whiskers represent variability outside of upper and lower quartiles.

As ENN B was consistently the most abundant ENN observed from in vitro culture extracts, the relative production of ENN B was assessed in situ from pea tissues and compared among *Fa*WT, Δ*esyn1*, and *ESYN1*_*OX* strains over the time course for both pea cultivars (cv. CDC Dakota and cv. CDC Meadow; Figure 8). An absence in accumulation of ENN B was observed for all Δ*esyn1* mutants in both *Fa*LH27 and *Fa*LH03 genetic backgrounds that corroborated observations in ENNs production from axenic cultures. Similarly, as compared to ENN production from axenic culture experiments, in situ ENN B accumulation was found to be greater in *Fa*LH27 *ESYN1_OX* mutants compared to *Fa*LH27 WT, where ENN B accumulation in *Fa*LH27 *ESYN1_OX* infected pea tissues accumulated steadily over the time course in both pea cultivars. Relative in situ accumulation of ENN B was found to be greater in pea tissues infected with *Fa*LH03 WT compared to *Fa*LH27 WT. As a general trend, in situ accumulation of ENN B was found to be greater in tissues of the susceptible pea cv. CDC Meadow compared to partially resistant cv. CDC Dakota for each of the time points sampled. From pea tissues for which ENN B was observed (most notably from tissues challenged with the FaLH27 *ESYN1-OX* strain), all ENNs previously found from in vitro culture extracts were observed to be produced in similar relative amounts (ENN B > ENN B1 > ENN A1 > ENN A).

## 3. Discussion

*F. avenaceum* has been found to produce numerous secondary metabolites [39], and, among these, ENNs were previously identified as aggressiveness factors in the *Fusarium–*potato tuber interaction [12]. Mycotoxins and other fungal secondary metabolites have been shown in numerous plant–pathogen interactions to interfere with host-defense responses and/or contribute to pathogenicity or disease severity acting as virulence or aggressiveness factors [37,38,50]. Pathogenicity is defined as the ability to cause disease, while factors of virulence or aggressiveness influence the degree of pathogenicity. In wheat, the *Fusarium* trichothecene mycotoxins are important aggressiveness factors [30], but the role of ENNs in FHB virulence has not been investigated. Furthermore, the role of mycotoxin production has not been examined in pea root rot, of which *F. avenaceum* is an important causal agent. 

In the work presented herein, the *ENNIATIN SYNTHASE* 1 (*ESYN1*) gene was targeted in two *F. avenaceum* isolates (*Fa*LH03 and *Fa*LH27) for gene disruption or constitutive overexpression, and their interaction with potato tubers, durum wheat spikes, and pea roots investigated. The *ESYN1* gene was not detected in the disruption mutants (∆*esyn1*), while a single copy of the *ESYN1* gene was found in the overexpression mutant (*ESYN1*_OX) (Figure S1), which expressed higher levels of *ESYN1* transcripts compared with *Fa*LH27 (Figure 1) and accumulated more ENNs (Figure 4), confirming in locus overexpression of the transgene. 

Potato tuber necrosis assays confirmed the results of Herrmann et al. [12] in their *ESYN1* gene disruption work, where it was found that loss of ENN production was co-related with lower tuber necrosis. The work herein further shows that *ESYN1* overexpression results in increased ENN production (shown in axenic cultures; Figure 4), as well as higher tuber necrosis compared with the wild-type progenitor strain (Figure 5). A higher degree of tuber necrosis was also observed in response to *Fa*LH03. ENN production was not directly investigated in potato tubers, and it is not known whether ENN accumulation could explain these differences between wild-type strains. Other undefined genetic and epigenetic factors may also contribute to this difference in aggressiveness.

Gene disruption in *Fusarium* has proven to be a powerful tool in the study of virulence factors in FHB [51,52,53]. Investigations of *F. graminearum tri5* mutants have demonstrated the importance of trichothecene mycotoxins in pathogenicity on wheat [30,54]. Even when trichothecene production is disrupted, *F. graminearum* continues to produce other classes of mycotoxins, such as butenolide, culmorum, and zearalenone [55,56], along with other factors associated with virulence [53], and the fungus is able to establish disease and cause necrosis in cereals. However, as demonstrated in studies of *tri5* mutants [30,57,58,59], trichothecenes were found to be necessary for the spread of *F. graminearum* through the wheat rachis. *F. avenaceum* does not produce trichothecenes [60] and is not as aggressive as *F. graminearum* on cereals [61]. While trichothecenes are necessary for *F. graminearum* to spread through the wheat rachis, there are perhaps other virulence factors present in *F. avenaceum* that may, at least in part, compensate for the absence of trichothecenes. Since ENNs are the most prominent *F. avenaceum* metabolite linked with FHB of wheat, it was anticipated that ENNs may be involved in disease aggressiveness in wheat spikes. This was not the case—no differences were observed in the disease development on durum wheat spikes cause by *F. avenaceum* strains either overexpressing or not expressing *ESYN1* (Figure 6).

There are no previous data on the role of mycotoxins in root rot of peas. Natural infection of field peas involves a complex of pathogens, presumably with each producing their own suite of secondary metabolites. ENNs are the most abundant metabolite produced in *Fa*LH03 and *Fa*LH27 (data not shown). Disruption of ENN production did not appear to influence pea emergence (Table 1) or disease severity (Figure 7) in controlled greenhouse experiments. Similarly, no difference was observed by increasing ENN production, as shown in the comparison of disease in pea plants inoculated with the wild-type progenitor strain. 

Since there are no prior reports of mycotoxin investigations in the pea root rot pathosystem, ENN accumulation was assessed in pea roots infected with WT and transgenic *F. avenaceum* strains (Figure 8). As expected, ENNs were not detected in Δ*esyn1* inoculated plants, whereas *Fa*LH27*ESYN1*_OX6 treatments led to a steady accumulation of ENNs (most pronouncedly ENN B) in pea roots and reached higher levels than either WT treatment. When comparing the WT treatments, a greater median in planta ENN B production was observed in *Fa*LH03 compared with *Fa*LH27 challenged pea roots of the susceptible cultivar (Meadow) at 14 and 18 DAS (Figure 8); however, no difference was observed in the severity of disease caused between these strains (Figure 7). ENN B also accumulated in higher amounts in the susceptible cv. CDC Meadow than in partially resistant cv. CDC Dakota, where comparatively little ENN B was detected. Since there was no observed correlation between disease severity and the ability of the fungus to produce ENNs, it is believed that the higher accumulation of ENN B in cv. CDC Meadow is related to a higher fungal biomass that one would expect to find in susceptible cultivars. 

Given that pea root rot is caused by a diverse group of pathogens, including but not limited to different *Fusarium* species [1,17], it could be that the accumulation of necrotizing factors facilitates this disease, where the specific toxin might be less important in establishing disease during and directly following pea seedling emergence. 

The importance of ENNs in pea root rot and FHB may be related to the interaction or competition of *F. avenaceum* with other fungi and/or mycotoxins. Willsey et al. [62] showed that *F. avenaceum* is always present with other pathogens in pea fields plagued with root rot. This species accumulates in roots later than the other pathogens and disease severity increases in its presence compared with single pathogen inoculations. In cereals, while *F. graminearum* is generally more aggressive in wheat than *F. avenaceum* and is the main causal agent of FHB, *F. avenaceum* is regularly detected in wheat spikes [4,31,32,63]. DON is the most common *Fusarium* mycotoxin identified in cereal grains [64], but it often co-occurs with ENNs. A survey of durum and bread wheat samples collected in Western Canada between 2010 and 2016 revealed that more than 70% were contaminated with both DON and enniatins [65]. 

It is possible that the enniatins produced by *F. avenaceum* will increase the toxicity/pathogenicity of co-occurring pathogens. In mammalian systems, the toxicity of ENN B has been found to increase by co-occurrence, in vitro, with other mycotoxins, depending on the concentrations tested [66]. Enniatins are known to form ion pores in cellular membranes and they can disrupt electrochemical potential of the electron transport chain in the mitochondria, thereby downregulating ATP production, which is required to drive the export of toxins from plant host cells [67]. Thus, the presence of enniatins inhibiting toxin export could increase the susceptibility of plant host cells to other mycotoxins. This could explain the reported increase in disease symptoms in peas co-contaminated with *F. avenaceum* and other root rot pathogens [62]. While ENN production by *F. avenaceum* does not appear to directly influence disease severity in pea roots or durum wheat spikes in controlled inoculation experiments, they may enhance disease pressure during interactions with pathogen complexes. 

*Fusarium* species possess a diverse suite of tools designed to overcome host defenses, including toxins, cell wall degrading enzymes, and effectors. The redundancy of these tools often makes it difficult to demonstrate that a specific gene is involved in virulence [68]. Thus, the role of enniatins could be at least partially compensated for by the action of other metabolites or enzymes involved in pathogenicity.

In summary, ENN production in *F. avenaceum* alone does not influence disease severity for FHB in durum wheat or root rot of peas in controlled greenhouse experiments. This is the first report of enniatin accumulation in *F. avenaceum* infected pea plants, and, while there was no correlation between ENN accumulation and disease severity, higher accumulation of ENN B was observed in the susceptible cultivar compared with the partially resistant one. While increased aggressiveness was observed in the tuber necrosis assay with increasing ENN production capabilities, it is not known whether the same would be true in disease assays of potato plants. For example, potatoes are also susceptible to *Fusarium* root rots and the interaction with the host plants may differ in isolated potato tubers compared with potato roots or even seeded tubers.

## 4. Materials and Methods 

### 4.1. F. avenaceum Strains and Spore Production

*F. avenaceum* strains DAOM242076 (*Fa*LH03) and DAOM242378 (*Fa*LH27), previously collected from Canadian wheat [39], were obtained from the Canadian Collection of Fungal Cultures (CCFC, Agriculture and Agri-Food Canada, Ottawa, ON, Canada). The fungus was grown on potato dextrose agar (PDA) with 50 µg mL^−1^ streptomycin at 25 °C. Mycelial plugs (10 mm) were collected from the margins of the PDA plates after six days of growth using a cork borer. Macroconidial spores were generated by either culturing one mycelial plug in 100 mL carboxymethylcellulose broth and purifying spores as previously described for *F. graminearum* [69] or by growth on 50% PDA plates for up to six days at 25 °C under fluorescent and black light to induce sporulation and collecting spores as previously described [70]. Spore concentrations were determined with a hemocytometer. 

### 4.2. Generation and Confirmation of ESYN1 Knockout and Overexpression Mutants

*ESYN1* disruption (Δ*esyn1*) and overexpression (*ESYN1_OX*) constructs were prepared in the pRF-HU2 and pRF-HU2E vectors, respectively, developed by Frandsen et al. [71]. Primers to amplify genomic DNA for cloning and transgenic validation were designed from the genome sequences of *Fa*LH03 and *Fa*LH27 (*Fa*LH03: JQGD00000000; *Fa*LH27: JQGE00000000) [39] (using *FAVG1_06797* in Genbank Accession: JPYM01000010.1 as a guide). The 5’ upstream region of *ESYN1* was amplified from *F. avenaceum* genomic DNA using forward (5’-AGTTCCCATCGCCACGGAG) and reverse (GGCTGATTTATGGATGATAAATG) primers and the 3’ upstream region using forward (5’-CGGCGAGCTGTATCAGAAACTAAG) and reverse (5’-GTCAAGAGGAAGATACGCCAGGTT) primers. pRF-HU2 was employed for targeted gene replacement with *HPH* for hygromycin resistance selection. pRF-HU2E enables in locus overexpression of a gene driven by the constitutive *Aspergillus nidulans* promoter (PgpdA) and also incorporates hygromycin resistance into the host. *Agrobacterium*-mediated transformation of *Fa*LH03 and *Fa*LH27 spores was carried out as previously described for *F. avenaceum* [72]. 

Putative disruptants and overexpression isolates were grown on PDA, genomic DNA extracted using the EZNA Fungal DNA Isolation Kit, and analyzed by PCR for presence or absence of the *ESYN1* gene and the hygromycin selection marker. Single spores were isolated, as described in the *Fusarium* laboratory manual [73], from all PCR-positive disruptants. Genomic DNA (10 µg) was digested with the restriction enzyme NcoI (New England Biolabs, Ipswich, MA, USA). The hybridization probes (amplified regions upstream and downstream of the disrupted gene using the above probes) were generated using the PCR DIG labeling kit and hybridizations done using the DIG Detection Kit (Roche Applied Science, Mississauga, ON, Canada), as per manufacturer’s instructions.

Mycelium was harvested from 1.5-day-old PDB cultures for RT-qPCR. For each of three biological replicates, mycelium from 3 × 50 mL Falcon Tubes, each with 20 mL PDB, was harvested and ground to a powder in a mortar and pestle with liquid nitrogen. Total RNA was isolated using an RNeasy® Mini Kit with an on-column DNase digestion (Qiagen, Montréal, QC, Canada), followed by an additional DNase digest with RNase-Free DNase I (Thermo Scientific^TM^, Waltham, MA, USA). Total RNA (1 µg) was combined with SuperScript III^TM^ Reverse Transcriptase (Invitrogen^TM^, Carlsbad, CA, USA), 50 µM oligo(dT)_20_ and 10 mM dNTPs in 20 µL cDNA synthesis reactions. Quantitative real-time PCR was carried out with PerfeCTa® SYBR® Green SuperMix, Low ROX^TM^ (Quantabio, Beverly, MA, USA) on a Quant Studio 6 Flex Real-Time PCR System (Applied Biosystems, Foster City, CA, USA). The reaction included an initial incubation at 50 °C (2 min) and then 95 °C (10 min), followed by forty cycles of 95 °C (15 s) and 54 °C (60 s). A melt curve was employed with the following steps: 95 °C (15 s), 60 °C (60 s), 95 °C (15 s). Four sets of primer pairs were designed to amplify products from transcripts of *ESYN1* (forward primer 5’-ACCGGACTAACGTCAACTGG; reverse primer 5’-CTCTCGCTCTGTCCGTAACC),and the following housekeeping genes *ACTIN BETA/GAMMA 1* (GenBank Accession MK547649; forward primer 5’-CCTGCTTGGAGATCCACATT; reverse primer 5’-CACTGCTCTTGCTCCTTCTT), *BETA-TUBULIN* (GenBank Accession: MK560857; forward primer 5’-CCAAATTGGTGCTGCTTTCT; reverse primer 5’-CTCGTTGAAGTAGACGCTCAT), and *TRANSLATION ELONGATION FACTOR 1-ALPHA* (GenBank Accession KU981027; forward primer 5’-GGAGGAGAAGACTCACCTTAAC; reverse primer 5’-GGTTCGCTTGTCGATACCA). Three technical replicates were employed for each of the three biological replicates along with the negative and positive controls. Positive controls and standard curve calculations were carried out as previously described [74]. Qiagen’s Relative Expression Software Tool (REST©, Montréal, QC, Canada) was utilized to normalize the data against the three housekeeping genes, and to calculate relative expression, from which log_2_ fold difference was calculated, and to compare different samples (*p* < 0.001) [75]. 

### 4.3. Spore Germination, Fungal Culturing, and Mycelial Growth Assays

Growth assays were carried out on each of the ∆*esyn1* and *ESYN1_OX* mutants and both WT progenitors (*Fa*LH03 and *Fa*LH27). For spore germination assays, Spezieller–Nährstoffar Agar (SNA) plates were used with a grid drawn on the back of each plate and the resulting squares (1 cm^2^ each) were spotted with 10 µL of 10^4^ spores mL^−1^. For each strain, ten squares per plate were spotted representing one experimental replication, where 2–3 replications were carried out, respectively. The plates were incubated at 27 °C for 20 h, after which the total number of germinated and ungerminated spores were counted under a microscope, and percent germination calculated.

Mycelial growth was compared on four different agar (20 g L^−1^) media: Potato Dextrose Agar (PDA; 2.4% Potato Dextrose Broth (Sigma, Oakville, ON, Canada)) Minimal Media (MM; 2 g NaNO_3_, 1 g KH_2_PO_4_, 0.5 g MgSO_4_·7H_2_O, 0.5 g KCl, 10 mg FeSO_4_·7H_2_O, 30 g sucrose and 0.2 mL Trace Elements [2.2 g ZnSO_4_·7H_2_O, 1.1 g H_3_BO_3_, 0.5 g MnCl_2_·4H_2_O, 0.5 g FeSO_4_·7H_2_O, 0.17 g CoCl_2_·6H_2_O, 0.16 g CuSO_4_·H_2_O, 0.15 g Na_2_MoO_4_·2H_2_O, and 5 g Na_4_EDTA in 100 mL distilled H_2_O] in 1 L distilled H_2_O), Yeast Extract Sucrose (YES; 20 g yeast extract, 150 g sucrose, and 0.5 g MgSO_4_·7H_2_O in 1 L distilled H_2_O), and Glucose Yeast Peptone (GYEP; 3 g NH_4_Cl, 2 g MgSO_4_·7H_2_O, 0.2 g FeSO_4_·7H_2_O, 2 g peptone, 2 g yeast extract, 2 g malt extract, and 20 g glucose in 1 L distilled H_2_O). Mycelia were collected from *F. avenaceum* WT and transgenic isolates grown on half strength PDA using an 18-gauge sterile needle. Using the needle, the mycelia were placed at the center of agar plates containing media, and incubated in the dark at 25 °C. After five days of incubation, radial growth of mycelia was measured in two perpendicular directions using a caliper. Five plates were used for each media and strain, and the experiment was repeated three times. Statistical analysis was done using a One-Way Analysis of Variance (ANOVA) followed by Tukey multiple comparison (Graphpad Prism software, San Diego, CA, USA). After 14 days of incubation, six agar plugs were sampled across the diameter of each colony, removed into 20 mL borosilicate glass vials, to which 20 mL of EtOAc was added and shaken at 225 rpm for 1 h on a rotary shaker. Resulting solvent extracts were then removed and dried down in a vacuum concentrator prior to resuspension in 1 mL of MeOH for UPLC-HRMS/CAD/DAD profiling.

Slant culturing was also performed using three different media broths: Czapek Yeast Autolysate (CYA) broth (3 g NaNO_3_, 1 g KH_2_PO_4_, 500 mg KCl, 500 mg MgSO_4_^.^7H_2_O, 10 mg FeSO_4_^.^7H_2_O, 5 g yeast extract (EMD), 30 g sucrose, and 1 mL trace element solution in 1 L Millipore H_2_O [Trace element solution: 1 g ZnSO_4_^.^7H_2_O and 500 mg CuSO_4_^.^5H_2_O in 100 mL Millipore H_2_O]); Mannitol Murashige & Skoog Salts (MMK2) broth (40 g mannitol, 5 g yeast extract (EMD), and 4.3 g Murashige & Skoog salts (Caisson Laboratories, Inc., Smithfield, UT, USA), in 1 L Millipore H_2_O); and Yeast Extract Sucrose (YES) broth (20 g yeast extract (EMD), 150 g sucrose, and 500 mg MgSO_4_^.^7H_2_O, in 1 L Millipore H_2_O). Fermentations were carried out in borosilicate glass culture tubes (50 mL) using 15 mL of broth medium, static, on a 30 °C inclination, in the dark, at 25 °C (four replicates per strain). After 14 days, mycelium mats were removed, frozen, and then extracted in 50 mL of EtOAc, shaken at 225 rpm for 1 h on a rotary shaker. Broths were extracted by partitioning with EtOAc (1:2, v:v), shaking at 225 rpm for 1 h on a rotary shaker, followed by removal of the EtOAc layer. Resulting solvent extracts were then removed and dried down in a vacuum concentrator prior to resuspension in 1 mL of MeOH for UPLC-HRMS/CAD/DAD profiling.

### 4.4. Potato Tuber Necrosis Assays

Tuber necrosis assays were employed as described by Herrmann et al. [11]. Tubers of the potato cultivar Russet Burbank were washed in running tap water, surface sterilized using 1% bleach for 5 min, rinsed three times with sterile water, and dried at room temperature for 15 min. Three layers of filter paper soaked in sterile water were placed in a petri dish to provide humidity. Approximately 6 mm thick slices were placed on the filter paper. Mycelial plugs (10 mm) of WT and mutant *F. avenaceum* isolates were collected from the margins of six-day-old PDA plates. Mycelial plugs were placed at the center of the tuber slice, mycelial side down. The tuber slices were weighed before and after the removal of the necrotic tissue that developed from *Fusarium* infection after six days of room temperature incubation in the dark. The difference in weight was used as a measure of the virulence of the WT versus ∆*esyn1* and *ESYN1_OX* mutants on the potato tissues. For each strain, ten potato slices were inoculated and the experiment was repeated three times.

### 4.5. FHB Assays in Durum Wheat 

*Fa*LH27 and corresponding ∆*esyn1* and *ESYN1_OX* isolates were compared for their ability to cause FHB in durum wheat. *Triticum turgidum* L. var. durum cultivar Langdon was grown in a growth cabinet (18 °C day/15 °C night; 16 h photoperiod) in 6.5-inch fiber pots of soil mix (75 PromixBx:24 Black Earth:1 Lime). One plant was seeded per pot, watered daily as needed and fertilized weekly. Powdery mildew was prevented by applying Sulphur Dust (Safer) weekly beginning at the 4–5 leaf stage; at four weeks before heading, a final treatment of EndTrust was applied (Corteva Agriscience). After trimming of awns, one spike per head was point-inoculated at mid-anthesis with 1000 spores of *F. avenaceum* in 5 µL of sterile water and heads loosely covered with a plastic bag. Plants were then misted for 1 min every hour for three days in a growth cabinet (25 °C/20 °C; 16 h photoperiod). After misting, plants remained in the growth cabinet with 80% humidity for 14 days. The experiment was repeated three times with ≥10 replicates (inoculated spikes) per treatment.

### 4.6. Pea Root Assays

The ability of the *F. avenaceum* WT and genetically modified isolates to infect pea roots was screened in two cultivars: a root rot susceptible field pea cultivar (cv. CDC Meadow) and a partially resistant dun-colored pea (cv. CDC Dakota). For mock inoculation, seeds were soaked in blank inoculum solution; for pathogen challenge, seeds were soaked in a macroconidal suspension (prepared as described above; 7500 macroconida mL^−1^) for each WT (*Fa*LH03 or *Fa*LH27) and ∆*esyn1* and *ESYN1_OX* isolates for 8 h. An inoculum concentration of 7500 macroconidia mL^−1^ was chosen based on preliminary experiments with the WT strains that indicated this was a discriminatory dose between the susceptible and partially resistant cultivars (data not shown). Seeds were then planted in individual 2-inch square pots in vermiculite, with two seeds per pot, and placed into an individual bag to prevent contamination between isolates. Vermiculite was wetted to run-off with sterile distilled water prior to seeding, and then at 3–4 d intervals after seeding to maintain adequate moisture for seed emergence. Once seedlings had emerged, plants were watered as needed, and vermiculite was allowed to dry out between watering periods. Plants were maintained in a greenhouse with a 16 h photoperiod and 21 °C day/18 °C night temperatures, at ambient relative humidity (65–70%). There were four replicate pots per treatment per time point, but not all plants emerged. Root rot severity was assessed 10, 14, 18, and 21 d after seeding (DAS). Roots were washed under running tap water and scored for infection symptoms (from 1 = healthy to 7 = tap root completely decayed, plant dead; Table 2) and lesion length (cm). For disease severity ratings, each pot was treated as a replicate unit, and the disease severity of the two plants per pot was averaged prior to performing statistical calculation of the means. Emergence percentage was calculated by counting all the plants that had emerged in all pots 10 days after planting, and dividing by the expected number of plants (two) per pot. Seeds that did not emerge but that had roots that were clearly colonized by *F. avenaceum* were given a rating of 7. The experiment was performed two times, and replicate nested within trial was treated as a random variable for statistical analysis. Treatment means were analyzed for each WT strain and its mutants separately by each evaluation day and cultivar using the REML method in JMP v14.0.

Plants were also harvested for metabolic profiling. Due to poor emergence, and to have enough plant material for mycotoxin analysis, two plants were pooled together and treated as a single replicate, and a minimum of six plants were harvested from each treatment (strain/mock vs. cultivar) at four time points (10, 14, 18, and 21 days) for further processing. At time of harvest, each plant was processed individually to normalize effects of sample handling. Plants were removed from the vermiculite substrate and washed briefly under running tap water to remove any adhering vermiculite, and blotted dry on filter paper. The lesion length was measured and then a 2-cm length of tap-root that included the point of seed attachment (seed was removed if still attached) and lesion was cut using a scalpel and flash frozen in liquid nitrogen and maintained in snap lock Eppendorf tubes at −80 °C. To reduce cross-contamination between samples, the scalpel was sterilized using 70% ethanol and flamed. Once all plants were harvested, frozen tissues were freeze dried for 48 h (LabConco 4.5 L Benchtop) and a 5 mm stainless steel bead that had been washed in acetone was added to each tube. Tissue was then ground to a fine powder using the TissueLyzer II for 60 s at maximum speed. For Meadow plants inoculated with *Fa*LH27*ESYN1_OX6* and *Fa*LH27∆*esyn1*_8, all roots had a rating of 7 at 18 and 22 days, at which point the root system was completely decayed so there was no material to collect.

In preparation for metabolic profiling of pea samples, 500 µL of extraction solvent (water:acetonitrile:acetic acid 20:79:1, *v*/*v*/*v* (with 40 µM caffeine as an internal standard)) was added to the polypropylene vials, containing milled root tissue samples. The samples were each vigorously vortexed for 10 s, followed by sonication for 4 min. The samples were subsequently extracted at 21 °C on a rotary shaker (at 225 rpm) for 90 min, protected from direct light exposure. The resulting extract was centrifuged for 2 min (at 12,000× *g*) to pellet the ground pea tissues; from which 150 µL of the extract supernatant was removed to amber glass HPLC vials (with glass inserts) and stored at −20 °C, within a 24 h period prior to UPLC-HRMS/CAD/DAD analysis.

### 4.7. Metabolic Profiling 

All high-resolution mass spectrometry (HRMS) data were collected using a LTQ Orbitrap XL Hybrid Ion Trap mass spectrometer (Thermo Scientific, Waltham, MA, USA) coupled to a Dionex Ultimate 3000 ultra-high-performance liquid chromatography (UHPLC) system (Thermo Scientific). For secondary metabolite profiling, chromatographic separation was achieved using a Kinetex C18 column (50 mm × 2.1 mm, 1.7 µm; Phenomenex, Torrance, CA, USA) maintained at 30 °C and a flow rate of 0.350 ml min^−1^. The mobile phase consisted of water with 0.1% formic acid (A) and acetonitrile containing 0.1% formic acid (B). The optimized 15 min mobile gradient consisted of mobile phase A, which was maintained at 95% for 0.5 min, before increasing to 95% solvent B over 4 min, and maintained at 95% solvent B for 3.5 min. For the remaining 7 min of the gradient, the column was allowed to equilibrate with 95% solvent A. The following parameters were employed for HRMS analysis (*m/z* 100–2000, resolution 30,000) in ESI+ mode: sheath gas (40), auxiliary gas (5), sweep gas (2), source voltage (4.0 kV), capillary temperature (320 °C), capillary voltage (35 V), and tube lens (100 V).

All “.RAW” data files including samples, MeOH blanks (run after every sixth sample), and medium controls, were processed using MZMine v2.51 (Cell Unit, Okinawa Institute of Science and Technology (OIST), Onna, Okinawa, Japan). For data preprocessing, mass detection was carried out with a noise cut-off level of 4.0 × 10^5^. Chromatographs were built using the ADAP algorithm with minimum group size set to 5, group intensity threshold set to 1.0 × 10^5^, and minimum highest intensity set to 1.0 × 10^6^. Chromatographs were deconvoluted using the Wavelets (ADAP) algorithm with signal/noise threshold set to 5, minimum feature height at 1.0 × 10^6^, coefficient/area threshold set to 90, and RT wavelet range set to max 0.2 min. Data were then cleared of isotopes, aligned, and converted into a data matrix of discriminate variables (based on RT and *m/z*) based on peak area measurements. Peaks were aligned using the Join Alignment function (with a *m/z* tolerance of 5.0 ppm or 0.005 *m/z* and a RT tolerance of 0.1 min with a 2:1 weight for *m/z* vs. RT). Gaps in the dataset where variables fell below the noise limit detection threshold were backfilled using a gap-filling algorithm (using a *m/z* tolerance of 5 ppm and RT tolerance of 0.05 min). Peak area values were normalized by dividing by the total ion current for each sample. Data complexity was reduced by summing variable peak areas across media conditions for each sample replicate to generate a representative secondary metabolite phenotype. Multivariate and univariate statistical analysis was performed in the R environment using the “muma” package and visualized using the ggplot2 package.

Chromatographic conditions were adapted to increase separation of enniatins for UPLC-HRMS/MS determination. The gradient initiated at 60% solvent B held for 1 min, increased to 95% over 2 min, held at 95% for 1.5 min, returned to starting conditions over 0.5 min, and allowed to equilibrate for 3.5 min. The HRMS was operated in ESI^+^ mode monitoring *m/z* 100–1000 using the following parameters: sheath gas (20), auxiliary gas (5), sweep gas (0), spray voltage (4.2 kV), capillary temperature (320 °C), capillary voltage (35 V), tube lens (100 V), maximum injection time (500 ms), and microscans (1). Targeted enniatin analog [M + H]+ pseudomolecular ions were called into the ion trap with isolation windows of 5 *m*/*z* (to allow for spectral accuracy determination of fragment ions) and MS2 scans were acquired using CID at 35 eV. Enniatin annotations were made by comparing retention times and MS/MS fragmentation patterns observed from commercial standards (Enniatin B, Sigma Aldrich, ≥95%) and with expected literature values and elution order [47]). MassWorks^TM^ software (v5.0.0, Cerno Bioscience) was used to improve spectral accuracy and confirm the molecular formulas of the daughter ions. The sCLIPS searches were performed in dynamic analysis mode with elements C, H, N, and O allowances set at minimum 1 and maximum 100. Charge was specified as 1, mass tolerance was set to 5 ppm and the profile mass range was −1.00 to 3.50 Da. For pea sample extracts, peak areas of enniatin B [M + H]+ ions were integrated and normalized using the caffeine internal standard using a processing method in Thermo XCalibur 2.2 software (Thermo Fisher Scientific Inc, Waltham, MA, USA).

## Figures and Tables

**Table 1:** Mean percent emergence of pea seeds, cultivars CDC Dakota or CDC Meadow, inoculated with *Fa*LH03, *Fa*LH27, and derived ∆*esyn1* and *ESYN1_OX* isolates. The control was mock-inoculated with blank inoculum solution, but was not included in the statistical analysis.

	Trial 1			Trial 2	
	Dakota	Meadow		Dakota	Meadow
***Fa*LH27**	40.6a †	29.2		81.2	6.2
***Fa*LH27Δ*esyn1*_2**	71.9ab	4.2		78.1	12.5
***Fa*LH27Δ*esyn1*_8**	81.2b	25.0		53.1	6.2
***Fa*LH27*ESYN1*_OX6**	71.9ab	41.7		62.5	21.9
SE *	6.48	10.38		8.87	7.50
***Fa*LH03**	81.2	29.2		50.0	0
***Fa*LH03Δ*esyn1*_2.1**	75.0	20.8		40.6	0
***Fa*LH03Δ*esyn1*_5.1**	87.5	12.5		50.0	9.4
***Fa*LH03Δ*esyn1_*6.1**	71.9	25.0		62.5	3.1
SE *	7.16	9.66		9.54	3.89
**Control**	75.0	79.2		100	75

* Standard error (SE) of the least square mean. † Means with the same letter are not significantly different according to Tukey–Kramer test (*p* < 0.05).

**Table 2:** Description of the visual rating scale (1–7) used to assess root rot severity (slightly modified from [76].

Rating	Lesion Description	Root Discolored (%)	Root Mass Reduction
1	0	0	0
2	Small (0.1–0.2 cm) reddish brown discoloration at point of seed attachment	0	0
3	Localized tap root/epicotyl lesions (0.5 to 1 cm) coalescing around ½ of tap root	10–20%	0
4	Lesions encircle tap root/epicotyl, 1–2 cm long	95%	5–10%
5	Tap root lesion 2–3 cm long, encircle root	100%	20–50%
6	Lesions > 3 cm long, root girdled	100%	50–80%
7	Total decay of tap root/epicotyl	Dead	Dead

## Supplementary Materials

The following are available online at https://www.mdpi.com/2076-0817/9/2/75/s1, Figure S1: FaLH27 disruption strategy and Southern analysis of Δe*syn1* modifications, Figure S2: Growth of Fusarium avenaceum FaLH27 and derived *Δesyn*1and *ESYN1*_OX isolates on potato dextrose agar (PDA), minimal media (MM), glucose yeast peptone (GYEP), and yeast extract sucrose (YES), Figure S3: Potato necrosis assay of potato tuber slices (cultivar Russet Burbank) inoculated with 10 mm mycelial plugs of FaLH27 and derived *Δesyn*1and *ESYN1*_OX isolates.
