# Supplementary material for: Enniatin Production Influences Fusarium avenaceum Virulence on Potato Tubers, but not on Durum Wheat or Peas
Source: Pathogens. 2020 Jan 21;9(2):75. doi: 10.3390/pathogens9020075 (PMC7168684; doi:10.3390/pathogens9020075)

Figure S1. FaLH27 disruption strategy and Southern analysis of Δ*esyn1* modifications. a) Schematic representation of the ESYN1 disruption and overexpression strategy. b) Southern analysis of SacII-digested DNA hybridized with HR1 (5') probe; c) Southern analysis of EcoRI-digested genomic DNA hybridized with HR2 (3') probe.

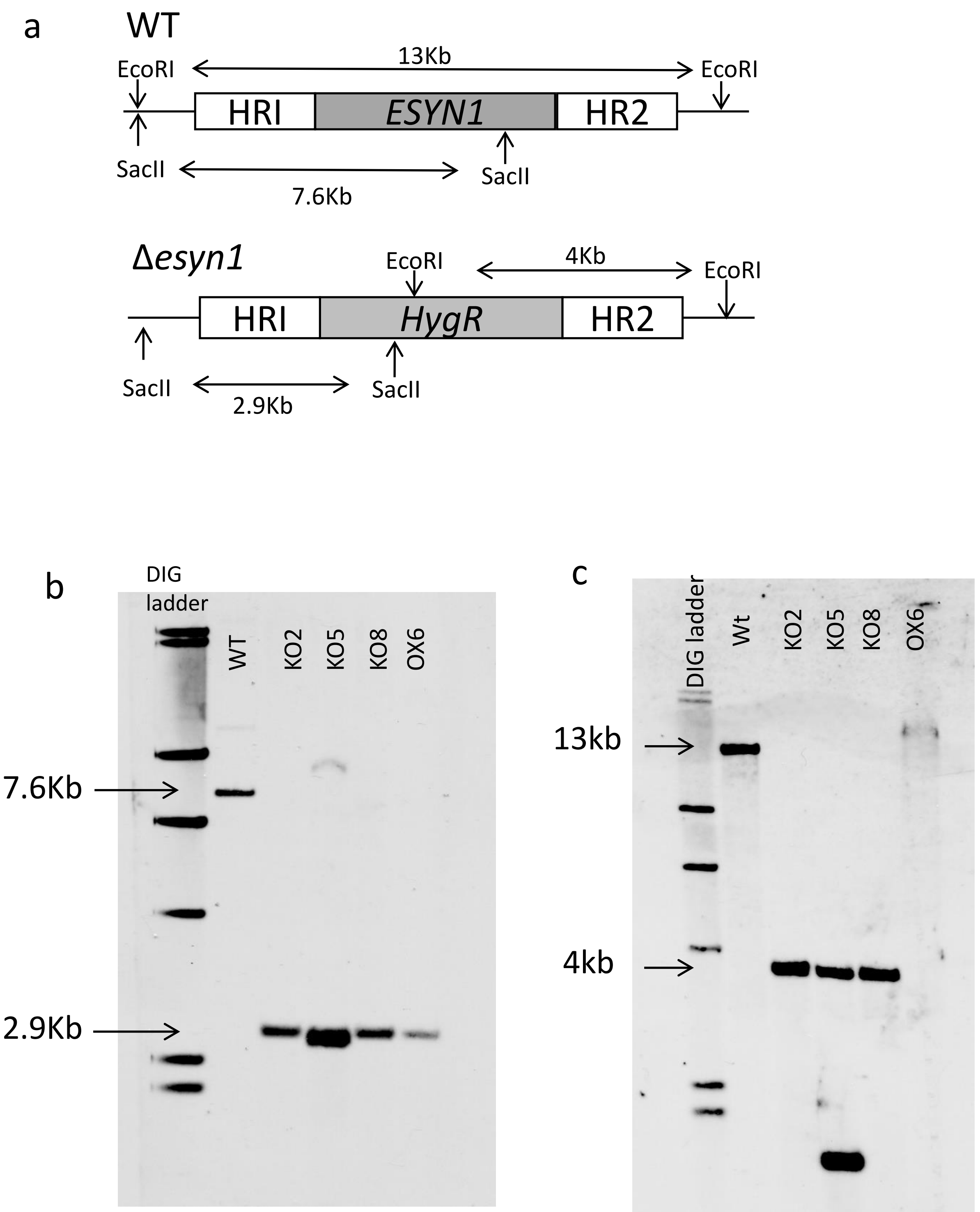

Figure S2. Growth of *Fusarium avenaceum Fa*LH27 and derived Δ*esyn1* and *ESYN1_OX* isolates on potato dextrose agar (PDA), minimal media (MM), glucose yeast peptone (GYEP) and yeast extract sucrose (YES)

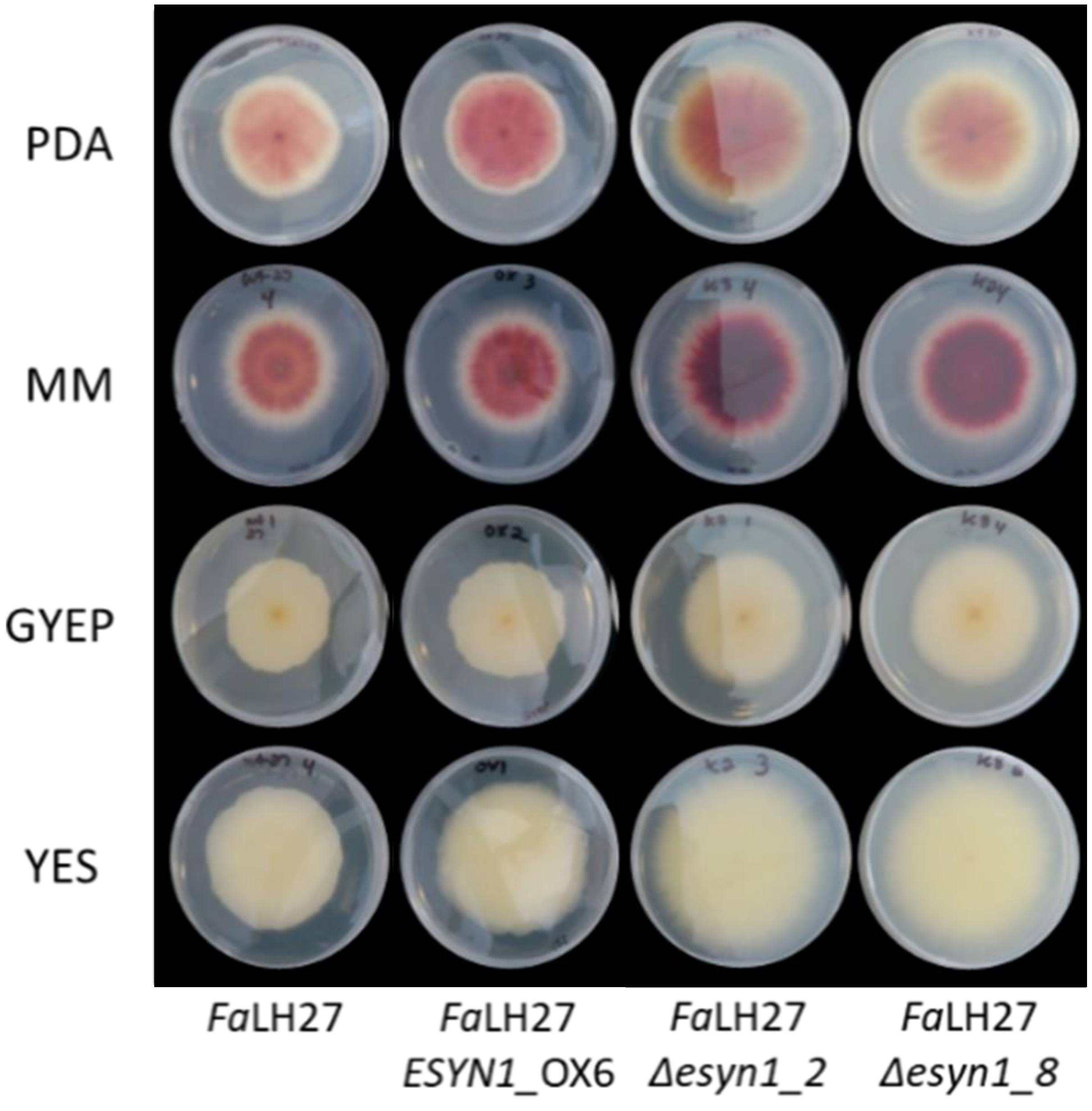

Figure S3. Potato necrosis assay of potato tuber slices (cultivar Russet Burbank) inoculated with 10 mm mycelial plugs of *Fa*LH27 and derived Δ*esyn1* and *ESYN1_OX* isolates. Necrosis was measured six days after incubation at room temperature in the dark. The left column shows necrosis of tuber flesh, and the right column shows tubers after necrotic tissue was removed from tuber slices.

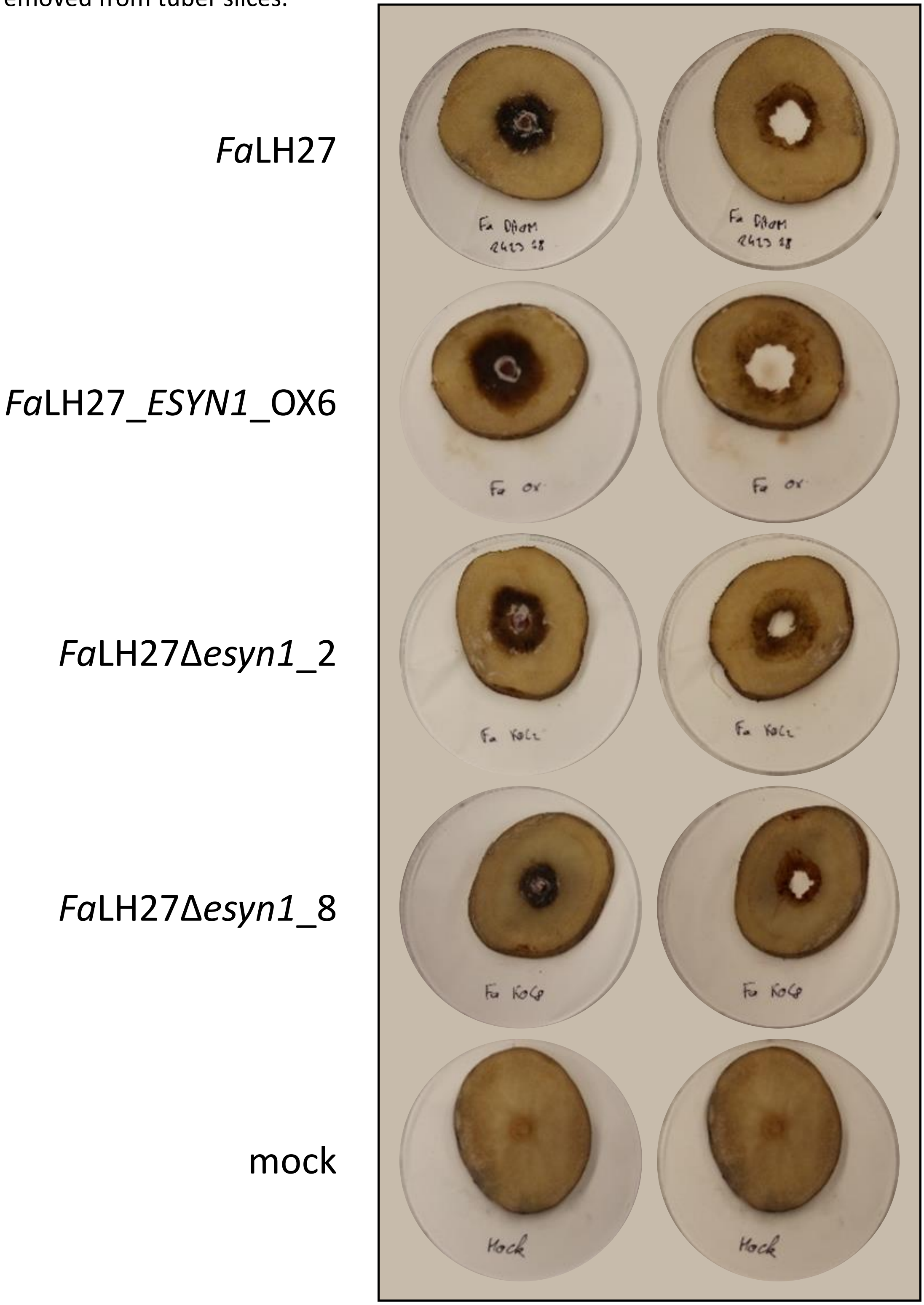

Supplement: Supplementary file 1 [file pathogens-09-00075-s001.pdf]
